# Active Ageing in Italy: A Systematic Review of National and Regional Policies

**DOI:** 10.3390/ijerph19010600

**Published:** 2022-01-05

**Authors:** Francesco Barbabella, Eralba Cela, Marco Socci, Davide Lucantoni, Marina Zannella, Andrea Principi

**Affiliations:** 1 Centre for Socio-Economic Research on Ageing, National Institute of Health and Science on Ageing (IRCCS INRCA), 60124 Ancona, Italy; f.barbabella@inrca.it (F.B.); d.lucantoni@inrca.it (D.L.); m.zannella@inrca.it (M.Z.); a.principi@inrca.it (A.P.); 2 Department of Social and Political Sciences, University of Milan, 20122 Milan, Italy; eralba.cela@unimi.it

**Keywords:** active ageing, older people, social participation, social inclusion, policy analysis, social rights, sustainability

## Abstract

Active ageing is defined as the process of optimizing opportunities for health, participation and security in order to enhance quality of life as people age. The design of active ageing policies intersects with different overarching societal challenges, especially ageing populations, social rights and sustainability. However, there are no previous attempts to review active ageing policies in the light of these challenges and the international policy objectives and targets that are guiding the international community. The aim of this study is to systematically identify, review and analyse all national and regional policies on active ageing adopted in Italy, by applying a conceptual framework derived from main international policy initiatives in the three areas. The research was conducted in two stages. First, a case study analysis was carried out per each relevant national institution and regional government. Standardised interviews were combined with policy document search, selection and analysis. Second, we performed a policy analysis in the light of a conceptual framework adopted. This latter was composed by nine policy domains, selected and integrated from principles and objectives of three overarching international frameworks on ageing—i.e., the Regional Implementation Strategy (RIS) commitments of the Madrid International Plan of Action on Ageing (MIPAA), social rights—i.e., the European Pillar of Social Rights and sustainability—i.e., the Sustainable Development Goals (SDGs) of the Agenda 2030 for Sustainable Development. Results pointed out that out of the identified nine policy fields, the major intervention areas by Italian policy makers concerned labour market participation, life-long learning, social and economic inequalities, health and well-being. Less attention had been given to issues such as gender and equal opportunities and sustainable cities. This systematic policy review is a milestone for understanding how active ageing policies contribute to address major societal challenges and what domains need further policy development.

## 1. Introduction

### 1.1. Active Ageing and Societal Challenges

Active ageing (AA) has been conceptualised as one of the possible policy responses to the ageing of the European population, a demographic trend that poses serious social and economic challenges to European societies. According to the World Health Organization (WHO) [1] (p. 12), “active ageing is the process of optimizing opportunities for health, participation and security to enhance quality of life as people age”. AA is expected to have positive effects on older people, since being active and engaged are assumed to benefit from healthier and happier lives, and societies at large, thanks to prolonged individual contribution in various domains of activities and a decreasing need for social security and public spending (especially in health and social care domains) [2,3]. A coherent and systematic adoption of AA within a country’s policy framework would imply a change of paradigm concerning older age, from a view of ageing as “passivity, decline and loss” to an understanding and promotion of the active roles older people may play in society by mobilising their personal resources [4].

Despite its widespread consideration and adoption worldwide, the AA concept received various criticisms both at a theoretical and practical level. First, even if AA is a broad concept that goes beyond promoting participation in employment, a productivist interpretation, focused on raising employment levels at all ages, initially prevailed at the policy level, mostly due the urgency to address demographic ageing and the related increase in pension and welfare costs [5]. Furthermore, the application of the AA concept sometimes lacked a person-centred approach, yet it is a risk to ignore that older people actually have agency (including their preferences and related choices) over their own ageing process and should not be pushed towards specific normative or model ageing [6].

Thus, there is a need to avoid a top-down approach when implementing the AA concept. Foster and Walker [7] identified some key points to consider when trying to turn the concept from theory to practice. For instance, attempts of its operationalisation at the policy level should pay attention to promoting meaningful activities that contribute to individual well-being. These opportunities should be offered to the heterogeneity of older people, not only those in better health and with more social and economic resources. Moreover, territorial and cultural diversity should be respected and considered by policies. This means that AA individual preferences and behaviours may be different according to the context. Lastly and most importantly, older people should be involved in co-decisional policy making [8] in order to avoid a top-down approach and to promote their agency.

AA has an important role to play in our understanding of and responses to population ageing [7], thus being a key to empowering individuals and improving their well-being along the life-course. This is particularly crucial if we consider the rising inequalities, insecurity and social risks faced by adult and older people in contemporary societies, a process known as “precarious ageing” [9]. The cumulative effects of precarity across the life-course—not only job-related but occurring in all life spheres (e.g., family changes, poor training opportunities, weaker social relations)—shape later life and bring additional social and economic inequalities [10]. Precarity is a politically induced condition [11], produced by institutional actors and decisions, and has been exacerbated in the last decades by declining social commitment and economic austerity and ultimately also by the COVID-19 pandemic. There is clearly a call for new and revised social rights and justice for older people and ageing individuals [12]. The concept of AA can be exploited in order to define new ways and options to support and empower people to better cope with contemporary risks and insecurities.

Furthermore, another major challenge for ageing societies is constituted by the necessity to assure a sustainable development in times when all countries face increasing environmental, social and economic risks. Sustainable development is defined as the development that “meets the needs of the present without compromising the ability of future generations to meet their own needs” [13]. Population ageing is a phenomenon intersecting various spheres of sustainability and impacting the way individuals contribute (e.g., economic development, unpaid care work, political participation and social capital) and need to be supported (e.g., poverty, social protection, health care and ageism) by societies during the life-course. From an AA perspective, “older persons must be recognized as the active agents of societal development in order to achieve truly transformative, inclusive and sustainable development outcomes” [14].

The design of AA policies is deeply interrelated with these overarching societal challenges, which have been particularly subject to public debate in the last few decades and put among priorities in the policy agenda. Ageing population, social rights and sustainability are key challenges for societies that require policy action. However, little is known in the literature about how AA policies contribute to facing these challenges. There are no previous attempts to review AA policies from this perspective or the policy response to such issues.

### 1.2. International Policy Frameworks

In the last two decades, three main international initiatives have tackled the abovementioned societal challenges and have incorporated specific policy objectives or targets, intersecting also (explicitly or implicitly) AA components.

In the area of ageing populations, the main initiative is represented by the Madrid International Plan of Action on Ageing (MIPAA), which was ratified by the General Assembly of the United Nations (UN) in 2002 [15]. The importance of the MIPAA relies on being the first global agreement that recognised the demographic and social transformations of population ageing as well as the key societal role of older people and their rights of secureness and empowerment in all the relevant ageing-related issues. In this perspective, ageing was considered a specific policy area to be addressed with innovative tools. Although the MIPAA is not a binding international agreement, but rather a political declaration, it has represented a turning point about how states look at ageing societies and longevity. Several principles—such as mainstreaming ageing, life-course perspective, social inclusion and combating ageism—were embedded in the MIPAA and have stimulated UN Member States to improve their policies in the field of ageing.

At the UN Economic Commission for Europe (UNECE) Ministerial Conference on Ageing in Berlin in 2002, a European Regional Implementation Strategy (RIS) for the MIPAA, was adopted [16]. The MIPAA/RIS included a set of 10 political commitments (Table 1), which aimed to adapt and focus the original MIPAA objectives into the social and economic peculiarities of the European region.

In the same year, the WHO contributed to the definition of AA and further advanced the idea of this concept by promoting a related multidimensional conceptualisation and policy framework [1]. The latter went beyond the mere health aspects and aimed to improve the general quality of life of ageing individuals, adopting a life-course approach and addressing health, social participation and security as main pillars [1]. Since then, AA has become an increasingly important concept within the EU policy framework, especially exploited in different initiatives such as the European Year of Active Ageing and Solidarity between Generations in 2012 [17], the European Innovation Partnership for Active and Healthy Ageing (EIPAHA) and the Green Paper on Ageing [18], which for a large part is focused on AA.

Concerning the domain of social rights, the most relevant advancement in recent years in the European area is constituted by the European Pillar of Social Rights [19]. It is a declaration aimed to foster fairness, inclusion and opportunities for social and economic growth in all Member States by setting 20 guiding principles (Table 2). An action plan was prepared by the European Commission for meeting the concrete delivery on the Pillar [20]. The European Pillar of Social Rights also reaffirms and builds on the concept of AA [21], although mostly at an implicit level and with some gaps [22,23,24].

In relation to sustainability, the WHO 69th World Health Assembly adopted the Global Strategy and Action Plan on Ageing and Health in 2016 [25]. The Global Strategy aimed to develop a renewed action plan that is aligned with the Sustainable Development Goals (SDGs) of the Agenda 2030 for Sustainable Development [26], a UN declaration. As recognised by the Global Strategy, ageing is an issue directly relevant for 15 out of 17 SDGs (Table 3), which address social and economic inequality, environmental issues, climate change and other societal challenges. Applying the SDGs in an ageing-related perspective is useful, since it allows trying to deal with some of the weaknesses identified in the operationalisation of the active ageing concept. For example, SDG 17 (partnership) also concerns participative bottom-un approach in a co-decisional way, which is important to the aim of avoiding a typical weakness of policy making in the field of active ageing, that is, a mainly top-down approach.

### 1.3. Aim of the Study

The ultimate goal of this study is to understand the contribution of current AA policies towards three major societal challenges—ageing populations, social rights and sustainability. More specifically, the aim is to systematically identify, review and analyse AA policies adopted in Italy at national and regional level by applying a conceptual framework derived from main international policy initiatives addressing those three challenges.

The specific research objectives are:

1. To understand whether, which and how many AA policies had been designed and implemented at the national and regional level;
2. To understand whether Italian AA policies are aligned, and to what extent they are, with key overarching international policy frameworks in order to identify areas of possible improvement.

### 1.4. The Italian Case

The present study focuses on AA policies in Italy. Although in Italy AA policies are obviously a component of welfare model and programs, there is a lack of cultural and political awareness on the theme, with few examples of dedicated national strategy. This is mostly due to the traditional weakness of public welfare programs. Literature on welfare/productive regimes classifies Italy among the Southern European countries, which fit the Mediterranean variant of the conservative-corporatist model, and having underdeveloped, or at least fragmented activation policy systems [27,28,29]. The Italian welfare model is also characterized by its familistic orientation, where the role of families is typically central in the provision of care and support to their older members [30,31], rather than considering them a resource for society according to the active ageing perspective.

However, in recent years, there has been an increased attention by Italian policy makers and stakeholders on the AA paradigm [32,33], with several emerging or established good practices. It has been demonstrated that the Italian population faces different experiences of AA due to institutional, policy and socio-economic differences between regions [34,35]. Furthermore, a first review of national and regional AA policies in 2016 showed an evolving policy context and interest at national and regional level [36,37]. However, public debate on AA has evolved slowly, due to the fact that in the last decades welfare reforms and changes focused mostly on other welfare needs and target groups (e.g., employment, pensions, health and social care) [38].

In this context, there is a wide knowledge gap concerning the types and range of AA policies in Italy [32]. Research did not produce yet a comprehensive recognition and analysis of Italian policies on AA, neither at national nor at regional level. A previous preliminary analysis of regional AA policies was conducted only in a selection of regions [36,37], and no further investigations were conducted until recently. Furthermore, no information or study is available concerning how AA policies address the three considered societal challenges and related international policy frameworks.

In the light of the abovementioned, the systematic review and analysis of all national and regional policies adopted in Italy will be carried out within the framework provided by the MIPAAs commitments, the principles of the European Pillar of Social Rights, and the SDGs of the 2030 Agenda for Sustainable Development. In this way, we aimed to cover and address the most important AA related aspects and to identify possible areas of improvements in terms of policy making. To the best of our knowledge, there are no previous studies (neither for Italy or for other countries) focusing on how AA policies contribute to overarching policies on ageing societies, social rights and sustainability.

## 2. Materials and Methods

This study was conducted within a wide national project (2019–2022) based on a multilevel and participatory coordination of AA policies, funded by the Department for Family Policies (DFP), a governmental office of the Italian Presidency of the Council of Ministers. The national project aims to develop, monitor and stimulate progress of AA policies, building national and regional stakeholder networks and improving awareness on AA among the relevant stakeholders and the population. The full project work plan and details are described elsewhere by Barbabella and colleagues [32].

The study was designed as a deductive thematic policy analysis aimed to discuss findings based on a conceptual framework. The focus of the study was on AA policies in accordance with the WHO definition [1] and concerned the ensemble of labour, social, educational and entertaining activities performed by older people and ageing individuals (i.e., AA during the life course). The link between each policy and AA could be either explicit or implicit, but still coherent with the definition of AA adopted for this study. In order to clarify that the focus should concern activation, which is an unexplored issue compared to other policy areas in Italy, some exclusion criteria were used. Our research excluded those policies regarding initiatives targeting older people merely as passive subjects, for instance those policies addressing only basic health (e.g., long-term care), social (e.g., home help) and economic needs (e.g., poverty).

The research did employ an inclusive, comprehensive and agency-oriented concept of AA. Our purpose was to investigate all policies that can enable people to engage, access and use resources for satisfying their own interests, attitudes and desires, despite any barriers they might face. Such a conceptualisation did not impose any specific, normative or reference model to AA, thus addressing and overcoming the most frequent concerns about the AA concept.

The research team aimed to conduct a systematic analysis, covering policies in the field of AA adopted by national and regional policy makers in Italy until end of 2019 and possible ongoing policy directions and acts under debate or approval. At the national level, research focused on legislative acts (and their implementation process) approved by the national parliament and the government—in this latter case, including interventions (e.g., decrees, regulations, guidelines) enacted by 14 relevant ministries and governmental offices (see the full list in Table 4).

At the regional level, research concerned all Italian regional governments, i.e., 19 Regions and 2 Autonomous Provinces (APs) (see the full list in Table 5). Additionally in this case, the study investigated regional laws, decrees and other policy acts promoting AA.

Main representative officers from all national and regional public administrations were already informed about the national project and the study, as they participated in the stakeholder network meeting aimed at presenting the project in June 2019 [32].

The current study was conducted in two phases. In the first phase (September 2019-February 2020), a case studies analysis, including a systematic data collection activity, took place by using multiple means. Each researcher of the project team was designated to carry out the case study in one or more (national or regional) governmental bodies. Standard protocol, topic guide and reporting template were adopted for standardising data collection in all institutions. The data collection process was conducted as follows:

1. The researcher carried out a desk review of policies and documentation publicly available through public web-repositories, literature, media and other channels;
2. The researcher asked the main representative(s) at the policy maker’s responsible office for AA (a) to provide the relevant policies and documentation on AA and (b) to organise an interview or group interview with the officers who directly manage or were involved in the implementation and monitoring of AA policies;
3. The researcher double-checked and screened the long list of policies and selected only the relevant ones, according to the study conceptual framework and exclusion criteria;
4. An interview or group interview was conducted (in-site, or via phone when it was not possible) per each Region/AP in order to get more information and to assess the degree of implementation and proper operation of selected policies;
5. The researcher organised all data and findings in a single report, using a common template, with the active contribution of the representatives interviewed.

We identified, selected and analysed public policies concerning AA that were designed and eventually implemented by national and regional policy makers. All 35 individual case study reports are publicly available (in Italian) on the project website [39], together with a preliminary report on the findings (in Italian) from the case studies (Barbabella et al., 2020b) [40]. Each report highlighted the evolution of AA policies by the institutions involved.

This fieldwork ended in February 2020, just days before the start of the COVID-19 epidemic crisis in Italy. This means that only policies concerning the period before the pandemic were selected and reviewed.

In the second phase, researchers analysed all the AA policies identified in the light of the conceptual framework adopted, which was defined as an operational tool to distinguish policy domains as major themes relevant for AA and structure the findings. As clarified in the Introduction, the three key sources for building the conceptual framework were the MIPAA/RIS; the European Pillar of Social Rights (EPSR) and the SDGs of the Agenda 2030. The respective commitments, principles and goals were linked and cross-analysed in order to identify overlapping themes and original aspects. The MIPAA/RIS was taken as the main thematic reference, given the close purpose and sectorial relevance for AA. Only those themes explicitly relevant for AA and ageing individuals were selected (while major societal challenges or issues were excluded, as only an indirect link could be found with ageing issues). The final conceptual framework included the following nine themes, that in this study were applied in an AA perspective:

1. Mainstreaming ageing in all public policies (MIPAA/RIS 1)
2. Ensuring integration and participation of older people in the society (MIPAA/RIS 2, SDG 17, EPSR 19)
3. Adjusting social protection systems, combating inequalities and sustaining economic growth (MIPAA/RIS 3, 4, SDG 1, 10, EPSR 3, 12, 13, 14, 15, 20)
4. Enabling labour markets for population ageing (MIPAA/RIS 5, SDG 8, EPSR 5, 6, 7, 8, 9, 10)
5. Promoting life-long learning and education (MIPAA/RIS 6, SDG 4, EPSR 1)
6. Ensuring quality of life, independent living and health (MIPAA/RIS 7, SDG 3, EPSR 16, 17, 18)
7. Mainstreaming gender approach (MIPAA/RIS 8, SDG 5, EPSR 2)
8. Supporting informal care to older people and intergenerational solidarity (MIPAA/RIS 9, SDG 16, EPSR 11)
9. Supporting sustainable cities and communities (SDG 11)

The abovementioned conceptual framework was used for framing findings of the deductive thematic analysis [41,42] in the nine policy domains. The qualitative thematic analysis included a comprehensive review of all primary (i.e., interviews) and secondary data (i.e., policy documents, legislative acts), an identification of themes for each national and regional sets of policies and their organisation within the conceptual framework in a process driven by our research objectives and questions [43,44,45]. The presence, design and implementation aspects of AA policies in Italy were cross-analysed for each theme, and the results are provided for both the national and the regional levels.

The research team guaranteed the credibility of the qualitative research [46] conducted in this study by employing the following approach: prolonged engagement (e.g., researchers had a robust experience and were trained on AA and the other topics covered); persistent observation (e.g., direct interactions and interviews carried out by researchers with experts and policy officers) and peer debriefing (e.g., continuous discussion within the research team about the aim, scope, methods, results and implications of the research and continuous consultation with and feedback by the national stakeholder network on intermediate project outputs).

## 3. Results

In Italy, at the national level, there is no specific definition of older people in the legislative framework. In the social protection system, older people are sometimes referred to as a frail target group of specific policies (e.g., long-term care). Similarly, no national law addresses explicitly AA. Three proposals of framework laws have been submitted to the national parliament (in 2016 and in 2019), without reaching the debate phase. Therefore, AA is currently targeted by means of a variety of sectorial policies, monitored by competent ministries. The domains where national policies are mostly concentrated are: working life, family, social participation and inclusion and health.

**Figure 1:**
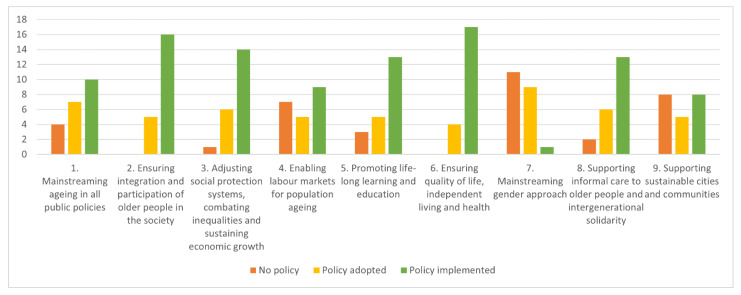
Number of Regions/APs which adopted and eventually implemented AA policies, per theme.

At the regional level, the situation is different. AA is progressively and explicitly considered by regional policy makers by means of dedicated framework regional laws and welfare programmes, as well as included in sectorial policies. The analysis identified regional AA policies (based on regional laws, regional government or council deliberations etc.), fitting the nine domains of the conceptual framework. The majority of Regions/APs developed policies are concentrated in specific domains (Figure 1), such as integration and social participation (Theme 2), promoting better health and well-being (Theme 6), supporting informal care and intergenerational solidarity (Theme 8) and adjusting social protection systems (Theme 3). Fewer Regions/APs have given attention to issues such as gender approach (Theme 7) and sustainable cities (Theme 9).

Below, we analyse the findings per theme, providing a summary and organisation of AA policies identified at the national and regional level.

### 3.1. Mainstreaming Ageing in All Public Policies

#### 3.1.1. National Level

AA can be recognised as a set of social rights, implicitly protected by the Italian Constitution (art. 2, 3 and 32) and linked to the principles of equality and dignity. The aim of the state is to remove any obstacle that hampers the development and social participation of individuals. Apart from this, there is no legislative framework at the national level that defines, regulates or promotes AA in general, and in a cross-cutting way across policy areas, as the mainstreaming ageing approach underlines. However, some initiatives have been taken for addressing broader issues and target groups in specific AA sectors.

First, AA recently became one of the key topics managed by the DFP, which, as already mentioned, launched and funded the national project for a multilevel and participatory coordination of AA policies [32], on which activities this study is based on. Second, the Ministry of Foreign Affairs and International Co-operation develops recommendations (together with relevant Departments) and contributes to the identification of measures for protecting and promoting the rights and dignity of older people, to be discussed by bodies such as the UN Open-ended Working Group on Ageing (OEWGA). Third, reforms of labour market, social security and social welfare policies have considered demographic ageing as a crucial phenomenon to address, with some examples of stakeholder involvement (a network for social protection and inclusive, interinstitutional groups on life-long learning). Fourth, the Ministry of Health incorporates the health aspects of AA in the concept of Health in All Policies (HiAP) by co-planning multilevel initiatives with other institutions. Fifth, the Department for Youth Policies and Universal Civil Service manages the universal civil service open to individuals of any age, addressing also the social needs of older people and promoting intergenerational exchanges.

Other national institutions have developed or funded only sporadic projects related to AA (although planned for the general population), such as the Ministry of Economy and Finances and the Ministry of Economic Development (on financial education for citizens), the Ministry of Agricultural, Food and Forestry Policies (on social farming) and the Ministry of Cultural Heritage and Activities and Tourism (on urban qualification).

#### 3.1.2. Regional Level

More than half of the Regions/APs (12 out of 21) have approved a framework law or general programmes on AA in a mainstreaming ageing perspective (Table 6). Most of these laws establish permanent interdepartmental boards on AA, involving representatives of relevant regional departments and offices. The importance of these initiatives concerns the possibility of supporting local communities and stakeholders for initiatives with a high social value, as well as a cultural change and awareness raising among the regional ecosystem. Veneto and Friuli-Venezia Giulia have implemented for some years a mechanism, which includes the definition of a strategic plan for AA regional priorities, the declination of operative annual plans with financial dedicated resources and calls for projects for public and non-profit organisations, and project monitoring. Mechanisms of stakeholder consultation are usually put in place for discussing and co-planning AA priorities. Other regional contexts have launched and implemented a strategic policy approach and concrete actions promoting AA without having a specific law on the topic but rather within a framework policy design operationalised in other acts and bills (it is the case of Emilia-Romagna and Umbria).

However, in most cases framework laws on AA are barely implemented yet (in 8 cases out of 10), due to very recent approval (most of them were launched in 2017–2019), missing planning or consultation bodies or even a scarce resource allocation.

Other Regions and APs have implemented sporadic policies on AA without proper coordination, as for instance Valle d’Aosta, AP of Bolzano, AP of Trento, Tuscany and Latium.

### 3.2. Ensuring Integration and Participation of Older People in the Society

#### 3.2.1. National Level

Social integration and participation is a theme strictly linked with the contribution of the Third Sector in the engaging, supporting and socialisation of ageing individuals. In Italy, a national reform of the Third Sector (L. 106/2016, D.lgs. 117/2017) recently made more homogenous the normative framework for volunteering, social promotion, cooperatives and non-profit organisations. Furthermore, the essential levels of social assistance (L. 328/2000)—social services and interventions due to citizens—are defined by national law and protected as social and civil rights by the Constitution (art. 117, as amended in 2001), although the implementation remains the responsibility of Regions, Provinces, municipalities and their associations. AA does not, however, appear as an explicit target, although initiatives carried out by the Third Sector and local public authorities usually adopt life-course approaches and address older people in order to improve their integration and participation.

The mentioned universal civil service is a national programme that provides funding, among the others, to projects focused on the engagement of older people in the society (public and non-profit entities from the universal civil service register can implement them).

Specific calls for projects for non-profit and public organisations were launched by the Ministry of Labour and Social Policies in 2016 (concerning intergenerational relationships and work-life balance) and by the DFP (on social integration and participation, as well as intergenerational support). Initiatives for the economic education and anti-fraud of older people and potentially vulnerable consumers were organised by the Ministry of Economic Development (D.D. 17 June 2019, D.D. 12 February 2019) and the Ministry of Internal Affairs. The Ministry of Agricultural, Food and Forestry Policies promotes the involvement of disadvantaged people—including older people—in social farming (L. 141/2015) both in strategic plans (e.g., National Agricultural Development Plan) and networks at the national and international level. More generally, the involvement of the Third Sector entities is sought also by various Ministries (e.g., Health, Foreign Affairs) for reaching narrower purposes, either for campaigns and initiatives or at consultation and planning level.

#### 3.2.2. Regional Level

All Regions and APs have systematic mechanisms in place for consulting and involving stakeholders (from civil society and other public organisations) in boards or bodies focused on social and/or health policies. In this respect, representatives from older people or organisations working on AA themes are always involved, as they usually play a significant role in the regional Third Sector ecosystem.

Some Regions (Veneto, Friuli-Venezia Giulia, Emilia-Romagna, Marche and Umbria) have already implemented further mechanisms specifically focused on AA, thanks to the stimulus of approved AA framework laws or similar programmes. Such mechanisms concern consulting boards with external stakeholders for planning regional AA policies and priorities, as well as interdepartmental bodies for assuring that AA is transversally considered in all policies and discussed with relevant stakeholders.

Other programmes for social participation and integration concern the involvement of older people as volunteers in social, cultural, recreational and sport activities (e.g., Liguria, D.G.R. 431/2016; Umbria, L.R. 11/2015; Marche, L.R. 3/2018). Those Regions with an approved framework law on AA have also included focuses on volunteering in old age and other social participation measures (including for instance the support to other people from disadvantaged groups and intergenerational activities).

### 3.3. Adjusting Social Protection Systems, Combating Inequalities and Sustaining Economic Growth

#### 3.3.1. National Level

A series of new national policies came into force in the period 2016–2019 in order to strengthen the universalistic approach for combating social exclusion and adapting the public social insurance system to societal, economic and family changes. In 2019, both a basic income scheme and a guaranteed minimum pension were introduced (L. 26/2019), replacing the previous “inclusion income” scheme (D.lgs. 147/2017). The new measures provide a monthly economic benefit (which integrates available income up to the poverty threshold) to the frailest groups (e.g., unemployed, socially excluded, homeless or retirees with low pensions). In the case of the basic income, beneficiaries are required to sign an agreement with public employment services (giving availability to accept job offers and to perform community service in the meantime) and eventually another with municipalities (in order to receive support to overcome possible social exclusion issues). However, the mechanisms for re-employment and social inclusion are not yet implemented as planned, and the measure still remains mostly as an economic allowance providing relief against poverty.

The Ministry of Labour and Social Policies established the Network for Social Protection and Inclusion, which coordinates a structural funding from multiple sources (funds for poverty, social policies and long-term care). Three-year plans are adopted for supporting the implementation of local services for the frailest social groups (children, adolescents and families, people with a disability, people with long-term care needs and people at risk of poverty and social exclusion). Further initiatives (by the Ministry of Economy and Finances, Ministry of Foreign Affairs and International Co-operation and DFP) have been carried out for advancing the debate (at national or international level) about the appropriateness of the pension system and its distributive effects, especially considering the increasing precariousness and low-wage jobs during the working life, as well as the number of older people with low pensions alongside changes occurred in social and family structures.

Furthermore, several educational initiatives have been put in place for empowering citizens of all ages to better handle savings, investments, pensions and insurances during their life-course (L. 15/2017), filling the gaps among the adult population about financial management (EDUFINCIPIA project by the Ministry of Education) and combating frauds against older people (protocol between the Ministry of Internal Affairs and regional capital cities). Further initiatives exploited social farming (L. 141/2015) for supporting sustainable growth and requalification of lands, targeted at the most vulnerable groups in society, including disadvantaged older people.

In terms of sustainability, Italy has been the first country among EU and G7 members to introduce 12 indicators of equal and sustainable well-being (BES) in the economic and financial planning (L. 163/2016), with implicit links with AA (e.g., life expectancy at birth, poverty rate and unemployment rate).

#### 3.3.2. Regional Level

The fight of social and economic inequalities is a fundamental theme in many regional policies that address the general population and disadvantaged groups. Additionally, under this theme, the main reference for Regions/APs is the framework law on social assistance (L. 328/2000). Regions/APs exploit mainly two operative tools for planning policies for social protection and improving equality: the Regional Social Plan, where policy priorities, strategies and interventions are set for adjusting the regional welfare, which uses its own resources and funding from the National Fund for Social Policies; the Regional Operational Programme (POR), which plans the allocation of European Structural and Investment Funds under the thematic priorities and regional societal needs. These tools are used for addressing the social protection needs of the general population, with a focus on specific groups that are more vulnerable.

In some cases, the POR was used also to plan the management of specific crisis contexts, such as in Marche for supporting citizens (including older people) in critical social and economic conditions after the dramatic earthquake in Centre Italy in 2016 (DGR 475/2018). Some Regions have designed and implemented initiatives for facilitating the access to services by vulnerable groups, including older people. In Molise (DGR 251/2019), “supportive ambulatories” were created in partnership between municipalities and local health authorities in order to bring primary care services to socially disadvantaged people and those living in rural areas. In Basilicata (DGR 970/2017), “community labs” offered guidance and social activities to vulnerable groups. The Umbria Region, through resources of the regional social fund, the regional health fund and the POR-ESF 2014–2020, developed specific actions on AA aimed for social and digital inclusion (e.g., through the “OpenUmbria” and “Gemma” projects) and for improving the quality of older people, as well as for combating the poverty of citizens of all ages. Furthermore, economic support is provided to people at risk of poverty or social exclusion, sometimes specifically tailored to older people such as in Campania (DGR 897/2018) or to family carers such as in Emilia-Romagna (L.R. 2/2014) and Abruzzo (L.R. 43/2016). Finally, some Regions/APs such as Friuli-Venezia Giulia have implemented a system for monitoring multiple facets of frailty in old age (over 75 years old), including both health and social needs, in order to refer them to appropriate health care and social services when needed.

Educational initiatives on household finances for older people, incentives to the employment of older workers and the involvement of older people in social farming projects are the most common initiatives implemented at the local level.

### 3.4. Enabling Labour Markets for Population Ageing

#### 3.4.1. National Level

AA policies addressing labour markets can be grouped under three areas. First, following reforms in the fields of the pension system (L. 92/2012) and labour market (L. 183/2014) and a general stagnation of national economy in recent years, the issues of long-term unemployment, precariousness in working life and mature-age unemployment became a key policy target. New unemployment benefits (e.g., NASPI, acronym of the benefit named “Nuova Assicurazione Sociale per l’Impiego”—New Social Insurance for Employment; ASDI, acronym of the benefit named “Assegno Sociale di Disoccupazione”—Unemployment Social Allowance) were created for supporting more people and for a longer time, together with incentives for hiring disadvantaged groups (including unemployed workers over 50 years) and pre-retirement support measures for certain categories (e.g., Ape, acronym of the measure named “Anticipo Pensionistico”—Pension Advance; Quota 100, which is the short-term specific pre-retirement measure which guarantees early retirement for those who worked at least 38 years and are at least 62 years old).

Second, a set of policies tackled the issue of vocational training during the life-course. The main purpose has been to improve the recognition of knowledge, abilities and competences during the working life, as an enabling factor for better chances in the labour market. This should occur by linking unemployment benefits to training, certification and job matchmaking, under the coordination of the National Agency for Active Labour Market Policies (although the implementation of such mechanisms is still suboptimal and fragmented across the country). Furthermore, a general framework exists for the validation and certification of job competences, educational titles and professional qualifications.

Third, some policies supporting better quality of work and age-friendly environments were pushed. New projects were supported by the Ministry of Health (D.Lgs. 81/2008) to promote employee welfare and, in particular, healthy lifestyles, including for instance the training of company physicians, application of ergonomics principles, the creation of company gyms and services. A call for a project by DFP funded (with 74 million Euros) new initiatives of company welfare for work-life conciliation. Other similar incentives were fostered by other means, for instance tax-savings on company welfare benefits, benefits for working parents and flexible working hours (D.I. 25 March 2016, D.I. 12 September 2017), as well as the introduction of smart working when possible (L. 81/2017). Finally, intergenerational exchange and solidarity in working contexts is supported by projects funded by DFP under the universal civil service initiative and by those funded under the social farming programme.

#### 3.4.2. Regional Level

Some Regions and APs have spent efforts in similar directions. On the one hand, they fund initiatives for supporting intergenerational training and valorising the experience of older workers. In Veneto, the AA framework law (L.R. 23/2017) forecasts the funding of projects aimed to transfer competences of older workers to the younger ones, including the transmission of ancient and artisanal crafts. An analogous provision exists for instance in Umbria for training digital skills of older people by students at schools. In Latium, intergenerational solidarity in the labour market was aimed to achieve both an increase of youth occupation and a prolongation of working lives of older workers by strengthening intergenerational knowledge transfer and support.

On the other hand, policies for improving work-life balance exist. Some Regions such as Campania (L.R. 33/2017), Abruzzo (L.R. 43/2016) and Emilia-Romagna (L.R. 2/2014) approved sectorial policies targeting working carers. Such policies promote more flexible working hours, as well as a recognition of care competences developed (which could be used also as credits for obtaining professional qualifications as nurse assistants or similar). In the AP of Bolzano, the Province promotes a certification for employers, which can be audited against company policy for work-life conciliation.

In Emilia-Romagna, a wide programme for health promotion (L.R. 19/2018) has, among its objectives, to improve safety and prevention at work. Other sporadic measures include support for disadvantaged workers. In AP of Trento, a programme (L.P. 32/1990) exists for sustaining older workers (over 53 for men and over 49 for women), who were dismissed or became unemployed, to reach minimum pension requirements by being engaged in community service (particularly in green, culture and care sectors). Social farming initiatives for older people are implemented in Marche (L.R. 21/2011), Umbria (L.R. 12/2015) and Sardinia (L.R. 11/2015).

### 3.5. Promoting Life-Long Learning and Education

#### 3.5.1. National Level

Education, training and learning during the life course are key components of AA. In Italy, these aspects are mostly addressed by programmes developed and coordinated by the Ministry of Education. First, the national Plan of Action for the Innovation of Adult Education (PAIDEIA) has been implemented, with a Working Group that promotes and coordinates the activity of the Provincial Centres for Adult Education (CPIA) and other educational programmes (such as the abovementioned EDUFINCIPIA project for financial management). CPIA are recognised as autonomous school institutions and aim to provide complementary training (for active citizenship and employment purposes, mainly) and research, tests and development on adult education.

Second, an Interinstitutional Board on Life-long Learning was established since 2012 with the aim of defining minimum standards and intervention strategies for life-long learning, as well as managing and monitoring local networks for education and training. In particular, the Ministry of Education promoted and involved the Board in the development of the National Plan for Guaranteeing Competences of the Adult Population, structured in five actions: fostering the participation of CPIA to building and maintaining local networks; sustaining mechanisms of competence guarantees for the adult population of labour age (especially for what concerns mathematical, linguistic, digital and transversal competences); strengthening centres for research, tests and development on adult education at a regional level; favouring the adoption of flexible training tools for adult education (including at distance/remote learning); promoting integrated educational pathways aimed to adults to obtain a professional qualification and/or degree, enabling a continuation of education in the third cycle (university and other educational opportunities).

Furthermore, some national initiatives targeted specific disadvantaged groups in need for training and education and were promoted by the Ministry of Education in collaboration with other competent Ministries: the regional plans for civic and linguistic training (with the Ministry of Internal Affairs); special programme for education and training in prisons and juvenile correction facilities (with the Ministry of Justice); projects for promoting financial education and combating frauds (with the Ministry of Internal Affairs, Ministry of Economy and Finances and Ministry of Economic Development); national project for mentoring and tutoring old entrepreneurs and newly resident young people in farming (with the Ministry of Agricultural, Food and Forestry Policies) and projects linked to foreign policy and international cooperation (with the Ministry of Foreign Affairs and International Co-operation).

#### 3.5.2. Regional Level

All Regions and APs have designed and implemented AA policies related to education, training and learning. Three main intervention areas can be recognised. First, many Regions explicitly promote and sustain the Universities of Third Age in their framework laws on AA (e.g., in Abruzzo), laws on health and social services (e.g., in Umbria), dedicated acts (e.g., L.R. 41/2017 in Friuli-Venezia Giulia) or through other means (e.g., the Demarchi Foundation, an in-house organisation of the AP of Trento). Universities of Third Age allow adults and older people to attend courses and workshops for cultural, social and entertaining purposes.

Second, a set of policies was found for improving the social inclusion and active citizenship of older people. Local programmes and projects are funded by Regions and APs specifically for AA or within broader social policies addressing older people and intergenerational solidarity. These policies sustain training activities for social participation, civic engagement, empowerment and recreational purposes. For instance: Veneto (L.R. 23/2017) funds AA projects about domestic and road safety, addictions, frauds and social involvement; Umbria (L.R. 11/2015) and Emilia-Romagna (D.G.R. 1809/2017) support initiatives for improving digital competences of older people; Molise (D.G.R. 659/2012) promotes projects on intergenerational knowledge exchange; Emilia-Romagna (L.R. 34/2002) supports the training of older volunteers in non-profit organisations; Piedmont (L.R. 11/2018) foster cultural education of older people.

Finally, some policies target the training and recognition of skills for better opportunities in the labour market. Some Regions developed instruments for family carers (Emilia-Romagna, L.R. 2/2014; Abruzzo, L.R. 43/2016; Campania, L.R. 33/2017), including the recognition of caring skills but also for finalising training and obtaining professional qualifications (e.g., healthcare workers), although only in Emilia-Romagna such provisions have been actually implemented.

### 3.6. Ensuring Quality of Life, Independent Living and Health

#### 3.6.1. National Level

Three main programmes by the Ministry of Health are in place in this respect. The first one is the National Prevention Plan, a recurrent 5-year plan addressing priorities and interventions for health prevention and promotion. It concerns the design of measures for changing healthy behaviours and lifestyles, primary prevention, early detection and diagnosis, self-care and integrated case management of chronic conditions. One of the objectives is explicitly to promote active and healthy ageing among older people (over 65 years) to increase healthy life expectancy in line with European strategic goals. The priorities of the National Prevention Plan are incorporated and applied by each Region/AP in their own multiannual Regional Prevention Plans.

The second national programme is the “Passi d’Argento” (Silver Steps) initiative, which is a surveillance programme for monitoring the health statuses among older population (over 65 years) and health prevention measures. Through a questionnaire administered to older people by healthcare and social workers, data were collected about determinants of physical, mental and social health, as well as the health and well-being of the older individuals. Based on these data and four main profiles of the target group (healthy with low risk, healthy but at risk, at risk of disability, with disability), recommendations are periodically drawn for improving health conditions of the older population.

A further programme is “Guadagnare Salute” (Gain Health), launched in 2007 as a strategy applying recommendations by the WHO and EU on health promotion. The ultimate goal is to reduce the weight of chronic conditions on both the healthcare system and society. The implementation of the programme is shared with Regions and APs and address four intervention areas: diet and nutrition; physical activity; smoke cessation and combating alcohol abuse.

In terms of physical activity, other specific initiatives have been developed: guidelines on physical activity for different age groups and in relation to specific physiological and physio-pathological statuses and population subgroups (promoted by an intersectoral board established by the General Direction for Health Prevention at the Ministry of Health) and projects for promoting sport and physical activity at all ages and during later life (conducted by the National Olympic Committee for Italy, CONI).

#### 3.6.2. Regional level

All Regions/APs have progressively incorporated aspects of AA in their regional health policies, particularly in the Regional Prevention Plans, Regional Health Plans, Regional and Local Social Plans and analogous strategic documents. These tools support a multiannual planning of interventions related to health and social care services, including health prevention, self-management of chronic conditions and physical activity as key components for adult and older population. In particular, physical activity is considered by regional decision makers as a key enabler for independence good health and quality of life. For instance, adapted physical activity (AFA) for older people is explicitly adopted by Tuscany (D.G.R. 903/2017), Marche (D.G.R. 887/2018) and Campania (D.G.R. 860/2015).

In other Regions/APs, group physical activity is not only a mean to maintain good health in old age, but also a social and recreational opportunity. In this sense, some examples are the following: in Friuli-Venezia Giulia (L.R. 22/2014) a project was launched for developing trekking pathways and walking groups for older people; in AP of Trento (D.G.P. 2412/2016), the TrentinoSalute 4.0 programme was created, with a competence centre which develops, tests and offers digital health solutions for the population (e.g., geocaching apps for group walking). The social component of quality of life is addressed also by regional initiatives sustaining social and recreational activities in centres, associations and clubs for older people. In almost all Regions/APs, there are funding instruments to support small local projects and activities organised by non-profit actors, particularly relevant especially in rural and mountain areas (e.g., in the AP of Bolzano, D.G.P. 332/2018).

### 3.7. Mainstreaming Gender Approach

#### 3.7.1. National Level

Among national policies, there is a general lack of recognition of the relevance of gender inequalities linked to AA and implications for personal, social and economic spheres. Few measures were developed by the Ministry of Labour and Social Affairs for addressing female occupation, company welfare and recognition of informal care. In the first area, mature female workers are recognised as a disadvantaged group with lower opportunities for re-employment. Therefore, some benefits were put in place for incentivising companies to hire female workers, especially in sectors where women are underrepresented (e.g., D.I. 13 October 2015). Concerning company welfare, some ministerial decrees (D.M. 25 March 2016 and 12 October 2017) promoted the adoption of company measures for favouring work-life conciliation, applying the last labour market reform and other legislation (e.g., L. 81/2017). Finally, family carers (who are largely composed by women) were recognised with the creation of a dedicated national fund (L. 205/2017), which was first applied in 2020.

#### 3.7.2. Regional Level

The review did not highlight regional policies specifically addressing gender inequalities in an AA perspective. In some cases, gender was included or mentioned in regional framework laws on AA (in Piedmont, Veneto, Marche, Abruzzo, Campania and Basilicata) as a relevant factor to address for guaranteeing equal opportunities. However, this remained a declaration of principles and was not applied concretely or explicitly.

The only Region concretely addressing the theme of mainstreaming gender approach was Emilia-Romagna, where the Regional Plan of Action for a Society of All Ages (PAR) (D.G.R. 2299/2004) and the Regional Social and Health Plan (PSSR) (D.G.R. 120/2017) both include strategic objectives and intervention areas to address gender inequalities in adult and old age.

### 3.8. Supporting Informal Care to Older People and Intergenerational Solidarity

#### 3.8.1. National Level

Informal care is an issue that has been progressively introduced in the Italian policy framework with ad hoc explicit provisions. First, the role and profile were defined in a national law (L. 205/2017), referring to family carers as those persons assisting and caring for a partner/spouse or close family member with long-term care needs (i.e., total invalidity and/or impossibility to walk alone). A National Fund was created, with an annual budget of 20–25 million Euros (currently managed by the DFP), to distribute resources to Regions for interventions and benefits for family carers. Furthermore, the National Prevention Plan by the Ministry of Health has incorporated a focus on home care and informal care, retrieved also from the previous National Plan for Dementia (2014) and National Plan for Chronic Diseases (2016). The integration of informal care and care provided by privately-hired care workers with formal health and social care services was set among the objectives. Another priority was to recognise and address hidden needs of family carers in order to reduce their social exclusion and discrimination.

Furthermore, following the Third National Conference on Family in 2017, the DFP launched a call for projects for public and non-profit organisations in order to support work-family conciliation, intergenerational solidarity, family-friendly social and economic contexts. The ultimate goal was to foster local interest and integrated networks between communities, families, employers and Third Sector organisations. In 2019, the Department for Youth Policies and Universal Civil Service funded also several projects on supporting older people in frail conditions, as well as on sport education with intergenerational recreational activities involving older people. Some national calls for projects in 2018 and 2019, managed by the Ministry of Economic Development, aimed to support the private sector in designing, producing or improving technological solutions for active and healthy ageing, especially those enabling older people to continue living in the community.

Finally, the abovementioned law on social farming (L. 141/2015) included the promotion of intergenerational relations in rural areas.

#### 3.8.2. Regional Level

In addition to ordinary legislation on social interventions and services for disadvantaged groups, many Regions/APs have developed policies addressing informal care and intergenerational solidarity. Some initiatives focus on intergenerational education and socialisation, with the involvement of older people in day care centres for children (such as the Elki centres in the AP of Bolzano, L.P. 8/2013), intergenerational knowledge exchange (such as digital literacy training by young to older people in Molise, D.G.R. 659/2012), safety in public spaces (such as older people monitoring crosswalks close to schools) and other (social, educational, cultural, recreational) activities (e.g., Latium, L.R. 11/2016; Basilicata, D.G.R. 970/2017).

Specific regional laws on family carers were approved in Emilia-Romagna (L.R. 2/2014), Abruzzo (L.R. 43/2016) and Campania (L.R. 33/2017). However, only Emilia-Romagna implemented such a policy in a comprehensive and consistent way and promoted information and training initiatives, psychological support, support groups, relief care, care allowances and work-family conciliation measures. In other regions, the role of family carers is sometimes recognised by Regional Prevention Plans or by Local Health Authorities (ASL), which can offer information and training. Furthermore, the AP of Bolzano encourages private organisations to get a specific audit (“famigliaelavoro”) for certifying and improving awareness about company policies for family carers and work-life balance.

### 3.9. Supporting Sustainable Cities and Communities

#### 3.9.1. National Level

Few national policies address this theme from different perspectives. On the one hand, some policies by the Ministry of Internal Affairs concern the safety of older people, with the implementation of educational campaigns by law enforcement bodies about personal protection from criminals, robberies and frauds at home and in public spaces. On the other hand, the Ministry of Health promotes active and healthy ageing by planning the creation of smart communities (exploiting domotic and assisted living technologies) and better urban spaces, as indicated in the Operative Health Plan—National Strategy for Smart Specialisation.

A further intervention area is the redevelopment of rural areas and urban spaces. The National Rural Development Programme by the Ministry of Agricultural, Food and Forestry Policies aimed to sustain urban redevelopment, whereas other measures (L. 158/2017) promoted the valorisation of small municipalities and old city centres. Broader policies regarding redevelopment are the National Bank of Rural Areas (L. 154/2016), which aims to redevelop abandoned rural areas and support socio-economic growth and the management and valorisation of real estate and lands confiscated from organised crime.

#### 3.9.2. Regional Level

Many Regions/APs have developed policies for supporting sustainable cities and communities, particularly under three intervention areas. First, most Regions/APs implement accompanying and social transport services for older people in order to facilitate access to health, social, educational, recreational and public services. Second, some redevelopment policies address the needs of people and older people living in rural and mountain areas, with the aim to improve living conditions and accessibility to those areas with better roads and opportunities for socio-economic growth (e.g., local initiatives in Valle d’Aosta, APs of Bolzano and Trento). Furthermore, Regions/APs usually offer incentives and discounts for older people to access public transports (buses, local trains and other transport means) and to facilitate their mobility (e.g., AP of Bolzano, D.G.P. 760/2016; AP of Trento, D.G.P. 62/2019; Friuli-Venezia Giulia, L.R. 22/2014).

## 4. Discussion

The study identified, reviewed and analysed a large set of national and regional AA policies in Italy. In general, we found a partial availability and alignment of Italian AA policies with international frameworks on ageing, social rights and sustainability. These policies are usually fragmented and lack a coherent strategy or framework at national level, but most Regions/APs developed own frameworks addressing AA in a multifaceted perspective. Reviewed policies are substantially aligned with the objectives and targets of MIPAA/RIS, European Pillar of Social Rights and SDGs (even if explicit references or links to these initiatives are scarce). However, some themes are not covered by policies, and gaps exist.

Concerning the first research objective (i.e., to understand whether, which and how many AA policies were designed and implemented), the policy review highlighted the absence of a national framework law or comprehensive national policies explicitly dedicated to AA in a mainstreaming ageing perspective. At the national level, rights and opportunities related to AA are implicitly spread through sectorial laws, programmes and initiatives. This is in line with the traditional design of the Italian welfare model, which is mostly based on categorical programmes and services [38] and has not yet incorporated a systematic life-course approach for AA. Older people constitute a social group that has been traditionally considered vulnerable and targeted by retirement, healthcare and social welfare programmes. The aim of the existing policies has mostly been to address individual needs in the later stages of life and not to address the empowerment and active citizenship of ageing individuals to improve their opportunities for health, security and participation in society.

Despite the lack of a national strategy or legislative framework dedicated to AA, we found several (implicit) references to AA in current sectorial policies. The areas where more efforts were spent were four: working life, family, social participation and inclusion, health. First, labour market and retirement policies have progressively tried to prolong working life, combat the unemployment of older workers (especially those aged 50+) and sporadically facilitate the work–retirement transition (with few options for accessing early retirement, especially in case of people who started working early in their lives or those with strenuous jobs), with less attention on providing other opportunities (e.g., empowering older individuals in senior entrepreneurship or other self-employed jobs).

Second, some policies have been put in place for supporting older people and ageing individuals considering also their family relations. Direct support (mostly monetary) was shaped by recent interventions for a universal basic income and pension (in order to combat poverty and social exclusion), for informal carers and for a network of family-work-community.

Furthermore, social participation and inclusion has been fostered mostly by the policies aimed to reinforce and reform the Third Sector. Non-governmental organisations, associations, unions and other civil society organisations constitute resilient networks for local projects and initiatives, which include strong synergies with local communities (e.g., universal civil service) and environments (e.g., social farming). The selected policies aim to sustain these networks and the creation of a proper “community welfare”, addressing also AA priorities.

Finally, quite a number of national policy interventions have been conducted for addressing health and well-being, including also ageing with autonomy and dignity. Health promotion and prevention programmes on a national basis include, among the others, campaigns for the involvement of older people in light physical activity (individually or in groups).

Concerning the regional level, our analysis identified almost everywhere a positive progress towards an incorporation of AA in regional policies, although in the context of fragmentation amongst Regions/APs. Almost all Regions/APs have at least one sectorial policy promoting specific aspects of AA, such as life-long learning (e.g., Universities of Third Age), volunteering (e.g., civil service for older people), informal care (e.g., recognition and valorisation of informal carers) and health promotion (e.g., incentives for sport and physical activity.

Dynamic ecosystems exist where AA has been a major priority of social, educational, labour and cultural policies in the last years. Some Regions (such as Veneto, Friuli-Venezia Giulia, Emilia-Romagna and Umbria) have designed and implemented systematic and comprehensive policies, with significant resource allocations and monitoring plans for updates and reviews. In most cases, a main driver for the adoption of AA policies was the approval of a regional law (or analogous acts) on AA, defining target groups, intervention areas, consultation mechanisms and budgets.

Despite 12 out of 21 Regions/APs have a regional framework law (or analogous) on AA into force, in many cases the proper implementation was delayed, due to internal reasons such as the missing designation of officers or bodies with planning responsibility or the scarce resources allocated. However, even in these cases and where no regional law exists, it was possible to retrieve policy innovations on AA, dedicated for instance to digital training and inclusion, intergenerational solidarity, light physical activity, transmission of traditional crafts, social farming and others.

Moreover, it is important to note that Italy is one of the European countries with most Regions/APs recognised by the European Innovation Partnership on Active and Healthy Ageing (EIPAHA) as reference sites for active and healthy ageing. Thirteen out of twenty-one Regions/APs have obtained this status, which represents a step forward in improving awareness on AA within the regional institutions, as well as an opportunity to exchange and discuss good practices for active and healthy ageing with an international network of peer institutions and stakeholders.

Concerning the second research objective (i.e., to understand whether Italian AA policies are aligned, and to what extent, with key overarching international policy frameworks), the application of the conceptual framework based on nine thematic domains gave important indications.

First, Regions/APs with a comprehensive policy framework on AA (e.g., Emilia-Romagna, Veneto, Friuli-Venezia Giulia, Umbria) have more systematic approaches towards AA and substantially address most of the policy domains, including mainstreaming ageing in all public policies (domain no. 1). The implementation of operative tools for co-operation between departments/institutions and consultation/co-decision mechanisms with civil society stakeholders in the AA field are crucial for reaching appropriate awareness, capacity and impact. At the national level, a systematic approach towards AA is still missing, as there are only sporadic, explicit initiatives, with limited interinstitutional co-operation.

Second, most national institutions and Regions/APs sustain AA discourse and objectives at least implicitly in key social policy domains: integration and participation of older people in the society (domain no. 2), social protection, inequalities and economic growth (no. 3), inclusion in the labour market (no. 4), life-long learning and education (no. 5) and quality of life, independent living and health (no. 6). Each Region/AP has its own policy framework for what concerns health and social services, and national policy makers have also key competences (either for high-level planning and financing—such as for healthcare system and social protection measures or for direct intervention—such as for labour market and education) on these subjects. Policies addressing these domains are identifiable basically everywhere among the investigated institutions, certainly with different focuses and diversities. In some cases, AA policies—aimed to empower ageing individuals, enhance opportunities and reduce barriers for participation in the society—are somehow implicit and mixed in ordinary social welfare programmes, mostly targeting frail older people or those at risk of social exclusion.

Third, despite the widespread relevance of informal care by family members for older people and people with disability, only in few cases specific policies were put in place. At the national level, just sporadic and late initiatives were taken, whereas at regional level only few Regions/APs actually designed a policy supporting family carers (among them, only in Emilia-Romagna is the related law implemented with proper recognition and valorisation of this target group).

Finally, two important domains—mainstreaming gender approach (no. 7) and sustainable cities and communities (no. 9)—resulted with very few (if no) policies, both at the national and regional level. The need for improving AA policies for combating gender inequalities in Italy was also recently raised and demonstrated [47]. Certainly, initiatives for addressing equal opportunities and redevelopment of urban and rural environments exist to a certain extent. However, the review found that the link with AA is not clear and more efforts are needed by policy makers to adjust policies under these themes for addressing AA.

This study has some limitations to consider. The review involved systematically all policy makers at national level (ministries and governmental offices) that were deemed to possibly have links with AA and older people. We investigated most of the government ministries (10 out of 19), whereas few of them with portfolios (Defence, Justice, Infrastructure and Transports) and without portfolios (Relations with the Parliament, Public Administration, Regional Affairs and Autonomy, South Italy, European Affairs) were not covered in the study because of low relevance of their mission with AA themes. During the data collection period, a government crisis occurred and led to a new political government in September 2019, with a minor reorganisation of few ministries without portfolio. Some regional governments also went through elections. The main representative officers from national and regional administrations remained however in charge and the data collection proceeded without disruption.

Our analysis meant to investigate the presence and possible implementation of policies based on interviews and document review, with the limited possibility of assessing their actual effects and impacts. Furthermore, the conceptual framework adopted, as well as the key international policies selected to build it, should be considered as a list of policy priorities developed by the competent international community of policy makers. The comprehensiveness and adequacy of these sets of priorities for AA should not be taken for granted acritically.

Finally, the data collection was finalised by February 2020, just before the start of the COVID-19 epidemic in Italy. This study presents a state-of-the-art overview pre-pandemic, so all the following efforts and emergency initiatives put in place for facing the new circumstances, protect and activate older people during the crisis were not covered in this piece of research.

Despite such limitations, the study has at least three innovative aspects. First, we adopted a comprehensive definition of AA, which did not apply a productivist or normative approach and embraced a wide ranges of AA themes. Second, we present a systematic and comprehensive attempt to screen all AA policies in Italy so as to understand the progress of AA in the legislative framework and policy alignment with major international objectives such as the MIPAA/RIS, Agenda 2030 and the European Pillar of Social Rights. To the best of our knowledge, such a mapping exercise is the first attempt to review all AA policies in a country. Third, the study was carried out within the framework of a wider national project, jointly coordinated by the IRCCS INRCA and DFP [48]. The project carries out activities for a proactive policy design and recommendation based on an interactive style with representatives from the institutional/governmental level and the civil society [32]. The ultimate aim is to analyse, reformulate and translate knowledge about Italian AA policies for contributing to Evidence-Based Policy Making (EBPM) [49]. This effort is sustained by a large stakeholder network (including policy makers, civil society organisations and research institutions) for a joint discussion about AA priorities in Italy and for the promotion, network and exchange of successful AA experiences [50]. The policy analysis presented here greatly benefited from inputs and validation by this stakeholder network.

More generally, our work constitutes an important contribution to this policy design and consultation process [32]. Relying upon the knowledge produced by this state-of-the-art analysis (first task), the research team already produced a set of policy recommendations [51], discussed and validated with the stakeholder network in February and March 2021 (second task). Further actions and research are ongoing in order to support national and regional policy makers to design, monitor and implement new AA policies. In particular, another round of consultation was conducted in 2021 for analysing if and how the policy recommendations could be applied by each (national and regional) institution and identifying intervention areas for improving AA policies (third task). Individual reports were published in the project website [48], and the overarching report will be available in early 2022. Furthermore, implementation and monitoring activities (fourth task) are currently planned by IRCCS INRCA and DFP, together with a follow up initiative, in order to develop a proper national AA strategy and provide a long-term perspective to the current design and consultation process.

## 5. Conclusions

The study results shed a new light on AA policies in Italy by identifying and classifying policies according to a set of policy domains derived from main international frameworks on ageing, social rights and sustainability. To the best of our knowledge, this is the first study to systematically review all AA policies in a country and frame them in the light of international policy frameworks. No previous evidence is available in the AA literature, neither concerning Italy nor other countries.

By this analysis, we showed an overview of what types of policies have been addressed so far by national and regional policy makers and how they are aligned with the abovementioned international frameworks. The results showed areas of possible improvement in terms of policy making, and should be used by policy makers and researchers working on the cross-cutting area of AA, in Italy and in countries sharing similar traits—e.g., lack of a national AA strategy, general weakness of welfare programs and a rapidly ageing population. Furthermore, we also showed a methodology to perform this analysis systematically within a country.

It is evident that a coordinated approach towards “AA in all policies” is not yet present in Italy, and several gaps should be filled in the current national policy framework and in some Regions/APs. The integration of AA policies in a more coherent and systematic way should be a key priority for Italian policy makers, considering also the progressive demographic ageing of Italian society (among the strongest in Europe and in the world). The ageing population is a phenomenon reshaping the demographic, social and economic balance of the country. AA is one of the keys needed to appropriately address this challenge in order to provide more opportunities to individuals during their life-course and a better quality of life in old age.

Avoiding precarious lives and guaranteeing sustainable development and progress for all are overarching objectives requiring further efforts in the field of AA by Italian policy makers. The lack of interlinks and consideration of international policy frameworks (such as the MIPAA/RIS, European Pillar of Social Rights and SDGs) can be overcome by a cultural and political sensitivity towards individual themes and solutions. This is the case of most Italian national and regional government bodies, which managed to design and implement AA policies that somehow fit the international objectives and targets. However, there is a clear risk that Italian institutions fail to fully exploit the experiences, as well as the wide policy discussions and public debates, already generated by these frameworks and analogous ones. The knowledge base made available by this study should be used by national and regional decision makers as a reference and stimulus for reflection, debate and policy making on AA.

We do not question the possibility that national and regional policy makers have developed and implemented successful policy measures for the general population in the considered domains, such as labour market, social protection and health promotion. However, AA requires a deep restructure of the Italian welfare model for addressing AA needs during the life course and ultimately overcome the perpetuation of traditional structures of familistic support, pension-based welfare and dominance of old-age frailty discourses. International frameworks can be a support and should be used to improve policy directions, welfare programs and opportunities offered to older people and ageing individuals.

This should not be seen as a process imposing or pushing for a model or normative ageing. AA concept and policies have been criticised because sometimes the focus relies more on productive and economic-relevant activities (e.g., work) and less on empowerment and the agency of ageing individuals. In the view adopted here, AA is a mean to achieve better quality of life by increasing opportunities and resources for ageing individuals, in the context of a desirable better social inclusion, integration and cohesion.

Future research on AA in Italy and Europe should also tackle how the concept and policies need to be adapted to the “new normality”, i.e., the circumstances and lifestyles influenced by the COVID-19 pandemic and emergency policy measures implemented to face it. Today more than any moment in the recent past, the pandemic dramatically exposed the real health, social, economic and living conditions of older people, highlighting and exacerbating inequalities, precarity and exclusion of some social groups. AA should be possibly reframed in this context of an at-risk, ageing and digital society, studying new intersections, issues and solutions.

## Figures and Tables

**Table 1:** List of commitments of the MIPAA/RIS.

No.	Commitments
1	To mainstream ageing in all policy fields with the aim of bringing societies and economies into harmony with demographic change to achieve a society for all ages
2	To ensure full integration and participation of older persons in society
3	To promote equitable and sustainable economic growth in response to population ageing
4	To adjust social protection systems in response to demographic changes and their social and economic consequences
5	To enable labour markets to respond to the economic and social consequences of population ageing
6	To promote life-long learning and adapt the educational system in order to meet the changing economic, social and demographic conditions
7	To strive to ensure quality of life at all ages and maintain independent living including health and well-being
8	To mainstream a gender approach in an ageing society
9	To support families that provide care for older persons and promote intergenerational and intragenerational solidarity among their members
10	To promote the implementation and follow-up of the regional implementation strategy through regional co-operation

**Table 2:** List of principles of the European Pillar of Social Rights.

No.	Principles
	***Chapter I: Equal opportunities and access to the labour market***
1	Education, training and life-long learning
2	Gender equality
3	Equal opportunities
4	Active support to employment
	*C**hapter II: Fair working conditions***
5	Secure and adaptable employment
6	Wages
7	Information about employment conditions and protection in case of dismissals
8	Social dialogue and involvement of workers
9	Work-life balance
10	Healthy, safe and well-adapted work environment and data protection
	***Chapter III: Social protection and inclusion***
11	Childcare and support to children
12	Social protection
13	Unemployment benefits
14	Minimum income
15	Old age income and pensions
16	Health care
17	Inclusion of people with disabilities
18	Long-term care
19	Housing and assistance for the homeless
20	Access to essential services

**Table 3:** List of SDGs.

No.	SDGs
1	End poverty in all its forms everywhere
2	End hunger, achieve food security and improved nutrition and promote sustainable agriculture
3	Ensure healthy lives and promote well-being for all at all ages
4	Ensure inclusive and equitable quality education and promote lifelong learning opportunities for all
5	Achieve gender equality and empower all women and girls
6	Ensure availability and sustainable management of water and sanitation for all
7	Ensure access to affordable, reliable, sustainable and modern energy for all
8	Promote sustained, inclusive and sustainable economic growth, full and productive employment and decent work for all
9	Build resilient infrastructure, promote inclusive and sustainable industrialization and foster innovation
10	Reduce inequality within and among countries
11	Make cities and human settlements inclusive, safe, resilient and sustainable
12	Ensure sustainable consumption and production patterns
13	Take urgent action to combat climate change and its impacts
14	Conserve and sustainably use the oceans, seas and marine resources for sustainable development
15	Protect, restore and promote sustainable use of terrestrial ecosystems, sustainably manage forests, combat desertification and halt and reverse land degradation and halt biodiversity loss
16	Promote peaceful and inclusive societies for sustainable development, provide access to justice for all and build effective, accountable and inclusive institutions at all levels
17	Strengthen the means of implementation and revitalize the Global Partnership for Sustainable Development

**Table 4:** List of ministries and governmental offices covered.

Ministries	Governmental Offices
Ministry of Agricultural, Food and Forestry Policies	Department for Equal Opportunities
Ministry of Cultural Heritage and Activities and Tourism	Department for Family Policies
Ministry of Economic Development	Department for Youth Policies and Universal Civil Service
Ministry of Economy and Finances	Governmental Office for Sport
Ministry of Education	
Ministry of Environment, Land and Sea Protection	
Ministry of Foreign Affairs and International Co-operation	
Ministry of Health	
Ministry of Internal Affairs	
Ministry of Labour and Social Policies	

**Table 5:** List of Regions and APs covered.

Regions
***North-West***
Piedmont
Valle d’Aosta
Liguria
Lombardy
***North-East***
AP of Bolzano
AP of Trento
Veneto
Friuli-Venezia Giulia
Emilia-Romagna
***Centre***
Tuscany
Umbria
Marche
Latium
***South***
Abruzzo
Molise
Campania
Apulia
Basilicata
Calabria
***Islands***
Sicily
Sardinia

**Table 6:** List of regional framework laws or general programmes on AA.

Region/AP	Law/Programme	Level of Implementation
Piedmont	LR 17/2019	Low
Liguria	LR 48/2009	Low
Veneto	LR 23/2017	High
Friuli-Venezia Giulia	LR 22/2014	High
Emilia-Romagna	DGR 2299/2004	High
Umbria	LR 11/2015	High
Marche	LR 1/2019	Low
Abruzzo	LR 16/2016	Low
Campania	LR 2/2018	Low
Basilicata	LR 29/2017	Low
Calabria	LR 12/2018	Low
Apulia	LR 16/2019	Low

## Data Availability

The original dataset of case study reports analysed for the current study are publicly available (in Italian) in the online project repository: http://famiglia.governo.it/it/politiche-e-attivita/invecchiamento-attivo/progetto-di-coordinamento-nazionale/pubblicazioni-e-documenti/le-politiche-per-l-invecchiamento-attivo-in-italia-rapporti-relativi-ad-ogni-singola-amministrazione/ (accessed on 4 January 2022).
